# Oxidation of Au/Ag films by oxygen plasma: phase separation and generation of nanoporosity

**DOI:** 10.3762/bjnano.11.143

**Published:** 2020-10-22

**Authors:** Abdel-Aziz El Mel, Said A Mansour, Mujaheed Pasha, Atef Zekri, Janarthanan Ponraj, Akshath Shetty, Yousef Haik

**Affiliations:** 1 College of Science and Engineering, Hamad Bin Khalifa University, P.O. Box 34110, Doha, Qatar; 2 Qatar Environment and Energy Research Institute, Hamad Bin Khalifa University, Doha, Qatar; 3 Department of Mechanical and Industrial Engineering, Texas A & M University-Kingsville, TX 78363, USA

**Keywords:** metal alloys, nanoporous, oxygen plasma, silver, thin films

## Abstract

The oxidation of Au/Ag alloy thin films using radio-frequency oxygen plasma was studied in this work. It was demonstrated that there is a phase separation occurring between silver and gold. In addition, it was shown that the preferential oxidation of silver resulted in a solid-state diffusion of silver toward the surface where it oxidized and formed nanoporous microspheres. The gold phase remaining in the film exhibited nanoporosity due to the injected vacancies at the metal/silver oxide interface. Based on the scanning transmission electron microscopy analysis coupled with energy dispersive X-ray mapping a mechanism was proposed based on solid-state diffusion and the Kirkendall effect to explain the different steps occurring during the oxidation process.

## Introduction

**Figure 1:**
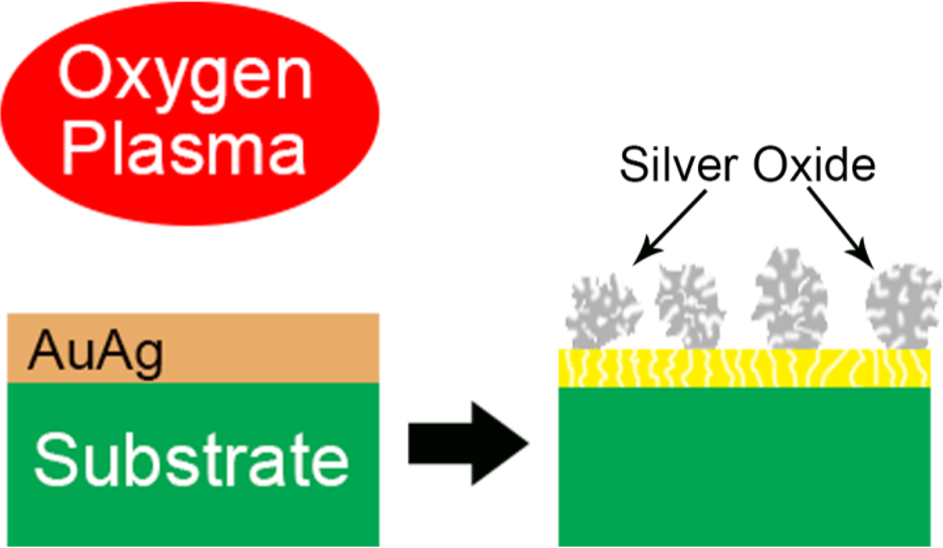
Schematic showing the procedure used in this work to study the oxidation of Au/Ag thin films by radio-frequency oxygen plasma, resulting in phase separation between gold and silver which led to the formation of nanoporous silver oxide microspheres at the top surface of the films.

Silver corrosion upon exposure to atomic oxygen is a phenomenon that was highly studied in the 1980s. At that time, the main aim was to avoid the degradation of silver interconnects and switches used in satellites navigating in the low earth orbit (LEO) [1–9]. Silver degradation related to this phenomenon was attributed to the strong chemical reaction between silver and atomic oxygen present in the LEO resulting in the transformation of the metallic silver into highly stressed and nanoporous silver oxide [1–2]. With the recent advancements in nanotechnology, this unique phenomenon received more attention since it can be used to generate nanoporous materials which are applicable in many areas, including drug delivery, biotechnology and sensor development. The interest in nanoporous materials originates from their special architecture and porosity, resulting in an extremely high specific surface area, at the nanometer scale, which can be suitable for many applications [10]. Details related to the silver oxidation process using either oxygen plasma or ozone have been previously reported [6–9]. Silver materials in various shapes and types were used to explore this process, including foils [11–12], films [13–17] and nanostructures [18–19]. So far, most of the published studies considered pure silver as the ideal model system to study the silver oxidation process and only a few have reported on the oxidation of silver alloys [15,20–21]. Gao et al. investigated the oxidation of Ag/Cu alloy by exposure to atomic oxygen. They demonstrated that the resistance of silver against corrosion by atomic oxygen was improved due to the presence of copper in the alloy [21]. From a fundamental point of view, gold/silver is a peculiar model system to study corrosion processes since gold is a noble metal. Interestingly, by using different types of Au/Ag nanoparticles, Lewis et al. showed how one can control and engineer, at the nanometer scale, the final shape and structure of gold/silver oxide nanostructures [20]. Starting from Au/Ag alloy nanospheres, they showed that gold/silver oxide core/shell nanospheres with a hollow interior could be obtained after oxidation using atomic oxygen. In this study we further explored the oxidation and phase separation events observed by Lewis et al. [20]. However, instead of using nanostructures as before, we used Au/Ag alloy thin films deposited by co-sputtering as the model system to study the oxidation process triggered by radio-frequency oxygen plasma (Figure 1). The oxidation and phase separation processes resulted in the formation of unique features, consisting of silver oxide nanoporous microspheres (Figure 1). Our observation was supported by various characterization techniques, including scanning electron microscopy (SEM), transmission electron microscopy (TEM) and X-ray diffraction spectroscopy (XRD). We conducted our study on Au/Ag films with different Ag concentrations but we presented only the results obtained for films with 75 atom % Ag as minor differences were noticed when other Ag concentrations were used (for films with higher Au content, the remaining gold layer after oxidation was thicker and denser). These minor differences were mentioned in the text whenever applicable.

## Results and Discussion

**Figure 2:**
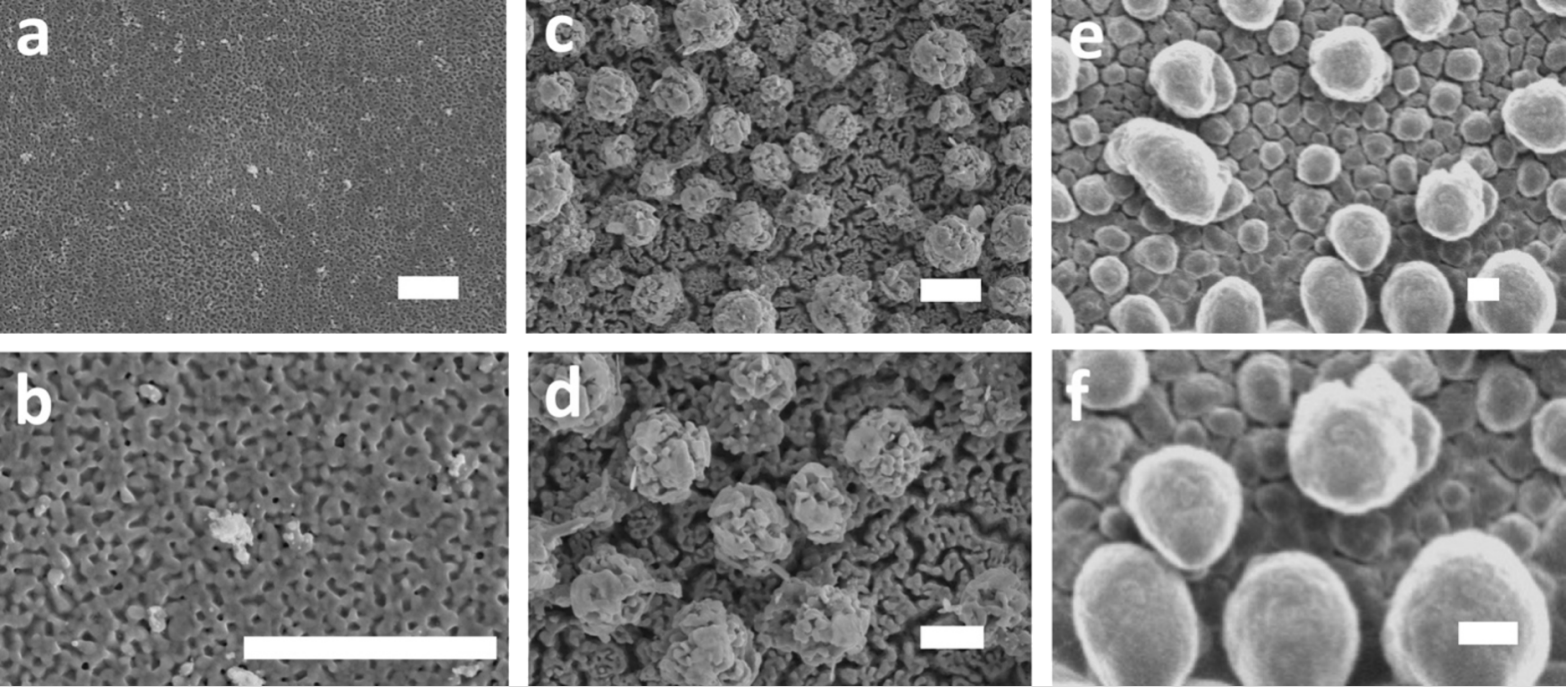
Top-view SEM images showing the morphological evolution happening at the surface of the films as a function of the oxidation time: (a, b) 3 min, (c, d) 15 min and (e, f) 30 min. For all the samples the electrical power was fixed at 100 W and the pressure was fixed at 3 Pa. Scale bar: 2 µm.

Figure 2 shows the morphological evolution at the surface of the Au/Ag films (75 and 25 atom % of Ag and Au, respectively) as a function of the oxidation time. After 3 min, small oxide grains start to form at the top surface of the films (Figure 2a,b). Increasing the oxidation time to 15 min results in the formation of nanoporous microspheres at the surface (Figure 2c,d). By using elemental energy dispersive X-ray (EDS) analysis, these microspheres were confirmed to be constituted of silver oxide. Interestingly, these microspheres do not cover the entire film surface but are rather randomly distributed over the surface. Moreover, when examining the surface an increase in porosity is noticed, probably as a consequence of the decrease in film density due to the migration of the oxidized silver toward the nanospheres (Figure 2d). By further increasing the oxidation time to 30 min, an increase in microsphere size and number is seen at the surface (Figure 2e,f).

**Figure 3:**
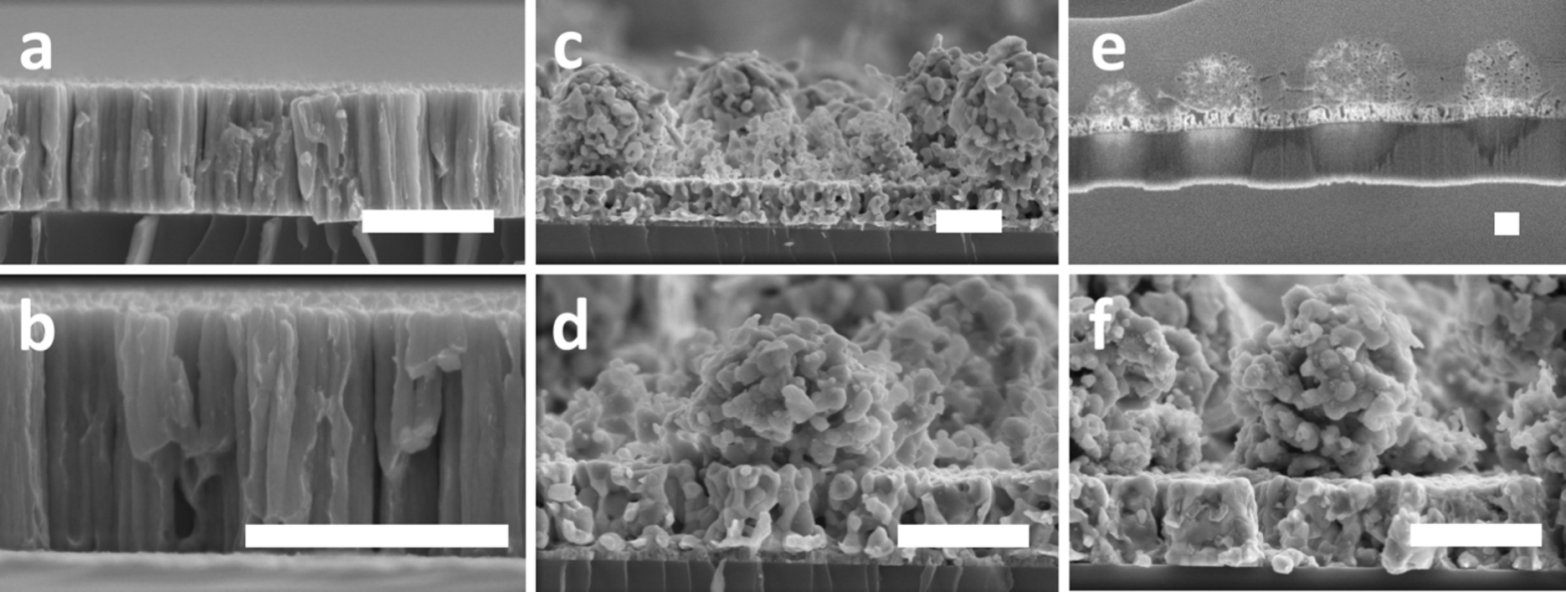
Cross-section SEM images showing the morphological evolution of the films as a function of the oxidation time: (a, b) 3 min, (c, d) 15 min and (e, f) 30 min. For all the samples the electrical power was fixed at 100 W and the pressure was fixed at 3 Pa. The SEM cross-section in panel (e) was prepared using FIB to verify whether the formed microspheres were hollow or not. Scale bar: 1 µm.

The evolution of film thickness was examined by SEM cross-section imaging (Figure 3). The results show that the as-grown films exhibit a columnar morphology typical of sputtered thin films at a low pressure and temperature. When the samples were submitted to oxidation for three minutes, there were no significant changes in film thickness or morphology in comparison to the control case (Figure 3a,b). However, when the oxidation time was increased to 15 min, the nanoporous microspheres started to form, as previously discussed, and a 30% decrease in film thickness was also observed (Figure 3c,d). The decrease in film thickness resulted from the phase separation event and the diffusion of silver toward the surface, which was also responsible for the generation of nanoporosity in the supporting films (Figure 3d). It is important to mention that the decrease in thickness becomes less prominent when Au/Ag alloy films with a higher Au concentration are used instead (data not shown). Overall, by further increasing the oxidation time the microspheres also increased in size (Figure 3e,f). To investigate whether the formed nanoporous microspheres have a hollow interior or not, a cross-section SEM specimen from the sample oxidized for 30 min was prepared using focused ion beam (FIB) (Figure 3e). According to the results, the microspheres were not hollow and the nanoporosity was not limited to the surface of the microspheres.

**Figure 4:**
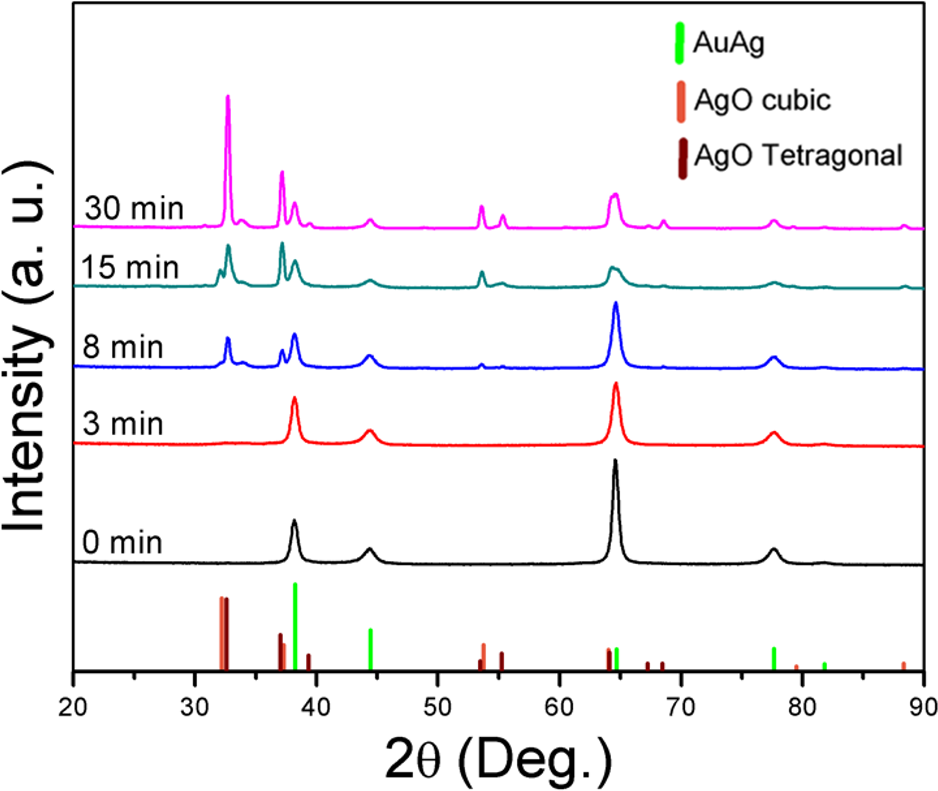
XRD patterns recorded on Au/Ag samples exposed to oxygen plasma for different durations. The powder diagrams represented by the columns were plotted using the following database files: Au/Ag JCDPS 01-071-9134; AgO cubic phase JCDPS 01-076-1489; AgO silver(I,III) oxide tetragonal phase JCDPS 01-084-1108.

To investigate the crystalline structure of the phases formed during the oxidation process, XRD patterns were recorded as a function of the oxidation time (Figure 4). The XRD pattern of the as-grown Au/Ag film (i.e., zero minutes of oxidation, black line) matches the cubic Au/Ag phase. Even though the Au/Ag film consisted of 75 atom % Ag, no crystalline silver phase was detected. After three minutes of oxidation no significant changes were observed in the XRD pattern (Figure 4, red line). This is probably due to the fact that both the size and amount of the oxidized features that are formed at the surface after such a short oxidation time are extremely small, as previously demonstrated by SEM (Figure 2a,b). When the oxidation time was increased to 8 min (Figure 4, blue line), new features that could be attributed to AgO silver appeared in the (I,III) oxide tetragonal phase diffraction diagram. After 15 min of oxidation (Figure 4, green line) an increase in the oxide phase signal was noticed. In addition, a AgO cubic phase was also detected after this oxidation period. However, the signal originating from the tetragonal phase was the dominant one. Prolonging the oxidation time to 30 min (Figure 4, pink line) led to a remarkable increase in the silver oxide phase intensity in comparison with the Au/Ag phase. This result indicates an increase in the oxide phase of the microspheres, which is consistent with the SEM results presented previously.

**Figure 5:**
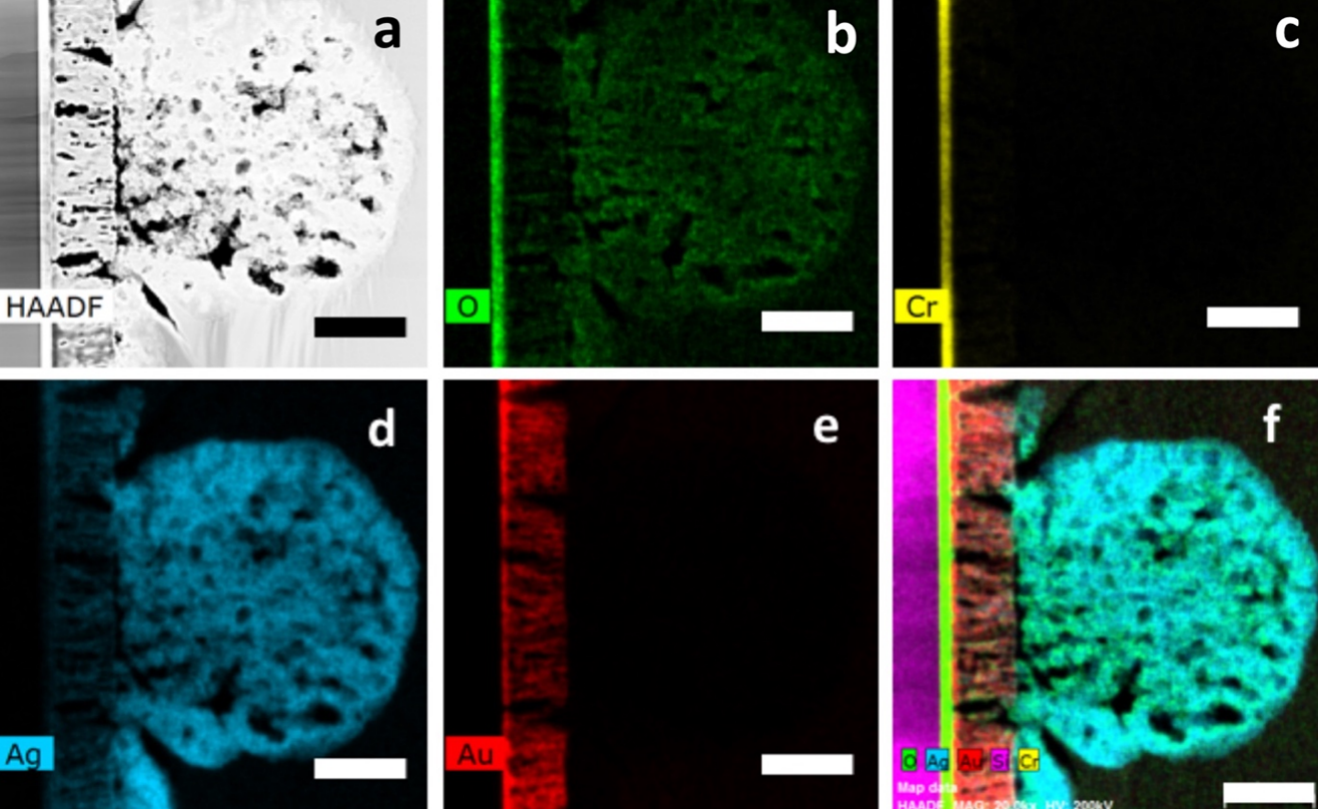
High-angle annular dark-field (HAADF) imaging (a) and color-coded STEM-EDS elemental mapping (b–e) showing the distribution of oxygen (green) (b), chromium (yellow) (c), silver (blue) (d) and gold (red) (e) after film oxidation for 30 min at 100 W. (F) RGB STEM-EDS image. Scale bar 100 nm.

The nanoporosity in the microspheres was further confirmed by cross-sectional scanning transmission electron microscopy (STEM) imaging performed on a sample oxidized for 30 min (Figure 5). STEM-EDS analysis showed that the microspheres were comprised of silver and oxygen, and it confirmed that no gold migrated toward the film surface.

**Figure 6:**
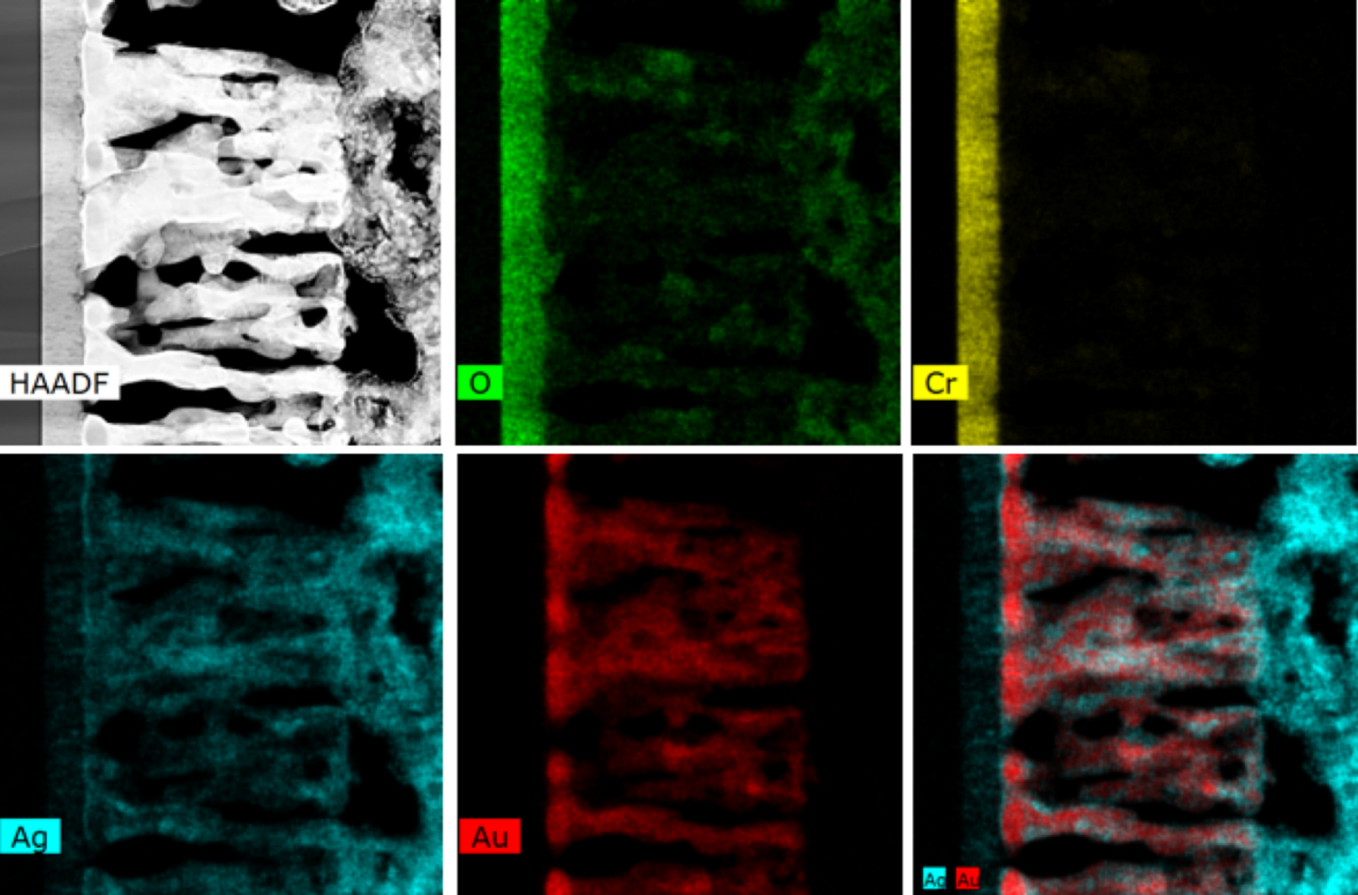
HAADF and EDS-STEM mapping of the Au/Ag film after oxidation revealing its porous structure.

According to the HAADF image in Figure 6, while the film maintained its columnar morphology, its nanocolumns became nanoporous, which is consistent with the cross-sectional SEM images of the same sample presented previously. The STEM-EDS elemental mapping showed that gold was present in the film. The EDS point analysis performed on the nanocolums showed a Au/Ag ratio of ≈3, which is significantly higher when compared with the same ratio before oxidation (Au/Ag ≈0.33 for the as-grown films). While oxygen was clearly present at the top layer of the silver oxide, its concentration was low inside the film. This indicates that the majority of the silver atoms diffused out of the film leaving behind a gold-rich nanoporous scaffold. The nanoporosity in the gold film probably resulted from the generation of vacancies within the film during the oxidation process and also due to the restructuring of gold at the nanometer scale via a surface diffusion mechanism.

Based on the STEM data (Figure 6) it is possible to conclude that silver underwent a solid-state diffusion mechanism to reach the upper surface of the film. In principle, this was an intriguing result since the plasma was kept at a low temperature during the film oxidation process which, in principle, should not trigger the silver migration. However, as discussed in our previous reports for the pure silver case [18–19], the local rise in the temperature at the nanometer scale resulted from the heat accumulation within the material during its exposure to the plasma, which probably led to the observed diffusion. Unfortunately, since the temperature rise was local it could not be experimentally measured.

**Figure 7:**
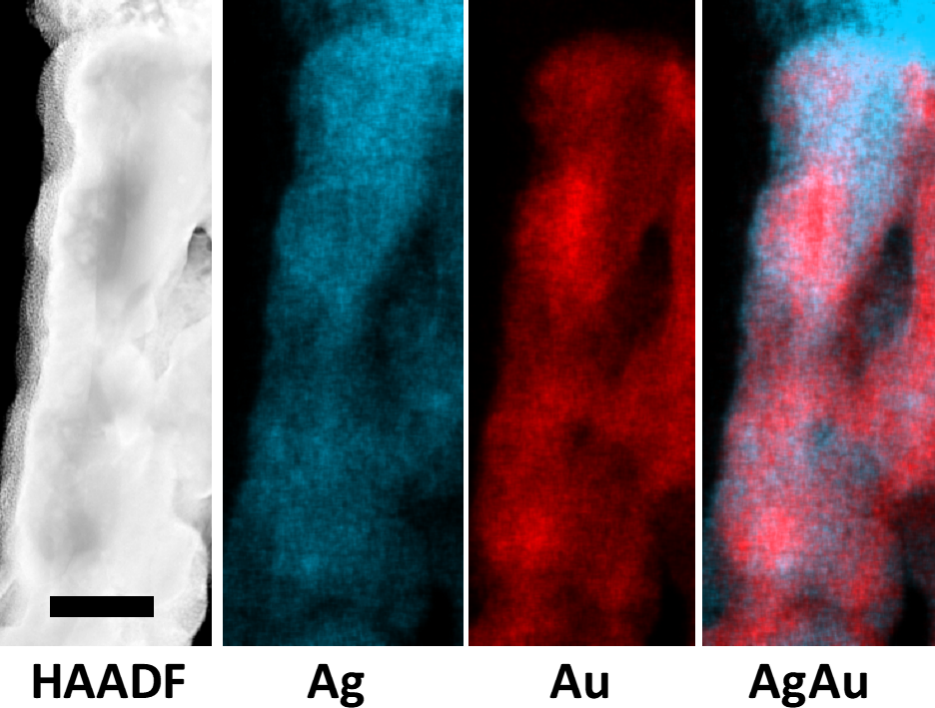
HAADF and EDS-STEM mapping of a column formed after 30 min of film oxidation. Scale bar: 100 nm.

STEM-EDS analysis performed on a single column after oxidation, showed a phase separation between gold and silver (Figure 7). HAADF and EDS-STEM image analysis revealed that the column was mainly constituted of gold surrounded with silver. This suggests that the migration of silver to the upper surface might have resulted from a grain-boundary diffusion mechanism. Indeed, in thin films silver diffusion happens either in volume (bulk diffusion) or through a grain boundary mechanism (short circuit diffusion) [22]. The latter is expected to be dominant as it exhibits a lower activation energy [23].

**Figure 8:**
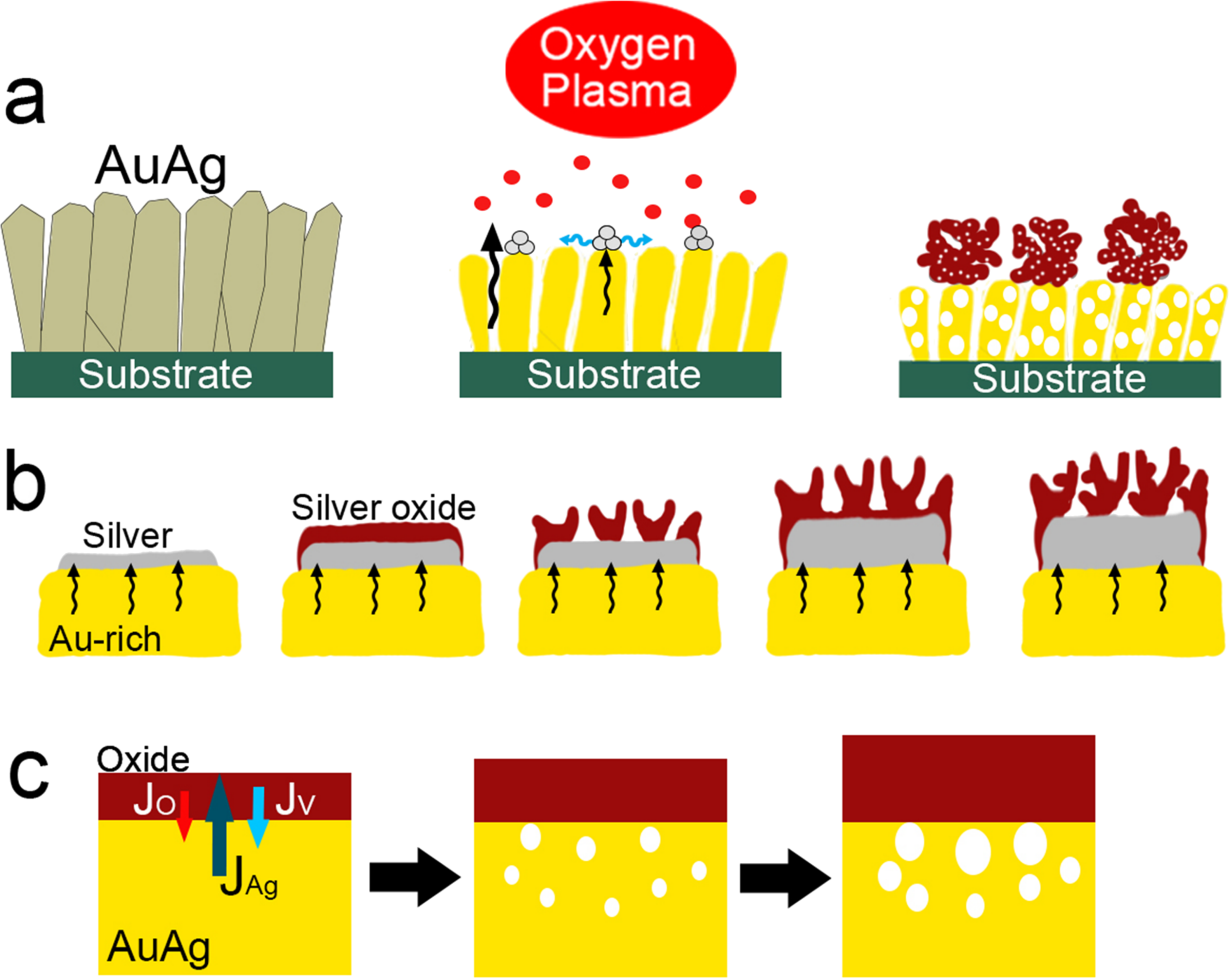
Schematic showing the different stages of the oxidation process. (a) Formation of nanoporous silver oxide microspheres at the top surface of the thin films. (b) Generation of nanoporosity due to the growth of silver oxide in a single column. (c) Generation of nanoporosity within the film due to an unbalanced diffusion rate between silver (outward diffusion) and oxygen (inward diffusion).

The following mechanism was proposed to explain the different steps of the oxidation process (Figure 8). In the early stages of oxidation, silver diffuses toward the surface where it nucleates and forms clusters (Figure 8a). When Ag reaches the film surface the diffusion is expected to become limited due to two factors: (1) the presence of oxygen which traps Ag during the oxidation process, resulting in a mobility decrease at the surface; and (2) the high porosity of the film which serves as a support for the surface diffusion of silver.

The clusters formed during the early stages of the process serve as nucleation sites which grow into larger spheres as the oxidation process evolves. The mechanism that generates nanoporosity inside the formed microspheres is similar to the one observed when pure silver is oxidized [18–19]. Briefly, in the beginning of the reaction, a highly stressed thin layer of Ag_2_O is formed on the silver phase according to the following reaction: 2Ag + O → Ag_2_O (Figure 8b). In the second stage of the reaction, the highly stressed oxidized layer cracks and peels in some areas, allowing for fresh patches of metallic silver to be exposed to the plasma oxygen. Subsequently, cracks and blisters are generated again in the newly formed oxide layer due to the high internal residual stress. The repeated oxidation and cracking events result in the transformation of the metallic silver into nanoporous silver oxide when it reaches the surface of the film. In parallel to the oxidation reaction, silver keeps diffusing from the alloy film toward the surface, feeding the metallic silver layer. The oxidation and cracking processes continue until the silver within the alloy film is totally consumed.

For the pure Ag film case [9], the oxide silver forms a continuous layer. However, for the alloy film case the silver oxide adopts a spherical shape due to the nanoporosity in the film surface, which serves as a substrate for the formation of the oxide phase. The presence of such a high porosity is expected to significantly reduce the silver mobility at the surface, posing difficulties to the formation of a continuous silver oxide layer. The formation of nanoporosity within the alloy film can be explained by the Kirkendall effect [24]. More precisely, as silver diffuses out of the alloy film, vacancies are injected into the metal/oxide interface and migrate within the fast-diffusing medium (represented here by the metal alloy in Figure 8c). As the oxidation process evolves, supersaturated vacancy clouds form within the metal alloy film. In order to minimize the energy of the system these vacancies tend to condensate and to form voids, which develop into nanopores in a later oxidation stage. The vacancy injection process continues until silver is completely extracted from the film and consumed.

## Conclusion

In summary, this work addresses the oxidation process of Au/Ag thin films by radio-frequency oxygen plasma. We have demonstrated that silver undergoes a preferential diffusion toward the film surface where it oxidizes and forms unique structures, consisting of nanoporous silver oxide microspheres. The co-existence of cubic silver oxide and tetragonal silver oxide was demonstrated using XRD. Furthermore, the STEM-EDS analysis showed that in addition to the silver oxidation at the surface there was a phase separation between gold and silver in the alloy film during the oxidation process. This phase separation led to the transformation of the Au/Ag alloy film into a gold-enriched nanoporous film. Based on the experimental results a mechanism was proposed to explain the entire oxidation process of Au/Ag alloy films by oxygen plasma, involving silver oxidation by atomic oxygen, solid-state diffusion in metal alloys as well as the Kirkendall effect. The nanoporous microspheres generated by the silver oxidation within the Au/Ag alloy film might have potential applications to the field of gas sensors and catalysis since those require nanoporous semiconductor materials with a high specific surface area.

## Experimental

The Au/Ag films were deposited by magnetron co-sputtering of gold and silver targets (99.99% in purity). The electrical power applied to the gold and silver targets was fixed to 25 and 100 W, respectively. This yielded Au/Ag films with 75 atom % and 25 atom % of silver and gold, respectively, as determined by EDS. The deposition time was 15 min, the deposition pressure was fixed at 10 mTorr and the distance between the substrate and the targets was ≈10 cm. All deposition processes were carried out on single crystal silicon wafers at a 30 rpm rotation speed and no intentional heating was applied to the substrate during the procedure. To ensure a proper adhesion of the Au/Ag alloy films, prior to each deposition a ≈100 nm thick chromium layer was deposited by sputtering of a chromium target (99.99% in purity), under pure argon atmosphere at 10 mT, for 12 min and at an electrical power of 100 W. The films were oxidized using radio-frequency oxygen plasma at 100 W and 3 Pa and the samples were placed onto a substrate holder located 10 cm away from the electrode.

SEM was performed on an FEI Versa3D instrument (Hillsboro, OR, USA) at an acceleration voltage of 5 kV and at a working distance of ≈6 mm. For EDS, a Bruker Quantax 800 detector (Billerica, MA, USA) was used at a fixed 10 kV acceleration voltage.

X-ray diffraction patterns were recorded in Bragg–Brentano geometry using a Rigaku SmarLab diffractometer (Tokyo, Japan). The voltage and the current of the X-ray generator were set to 45 kV and 40 mA, respectively. The signal was collected between 20° and 90° with a step size of 0.02° and at a speed of 1 deg/min.

STEM and STEM-EDS were performed on a TALOS (FEI, Hillsboro, Oregon, USA) field emission gun transmission electron microscope equipped with an FEI EDS detector and a high-angle annular dark-field (HAADF) detector operating at 200 kV. For qualitative elemental chemical analysis, ESPRIT software from Bruker was used. The cross-section film (thickness <100 nm) was prepared using a SEM/FIB Versa 3D dual beam instrument from FEI.
